# Supplementary material for: Estimating total human deaths from rabies: A statistical framework leveraging reports of rabies transmission from deceased organ and tissue donors
Source: PLoS Negl Trop Dis. 2026 Sep 21;20(9):e0014731. doi: 10.1371/journal.pntd.0014731 (PMC13592719; doi:10.1371/journal.pntd.0014731)
Supplement: Supplementary file 6 [file pntd.0014731.s005.zip › Result_B/Morocco/Figure_S3_Morocco.pdf]

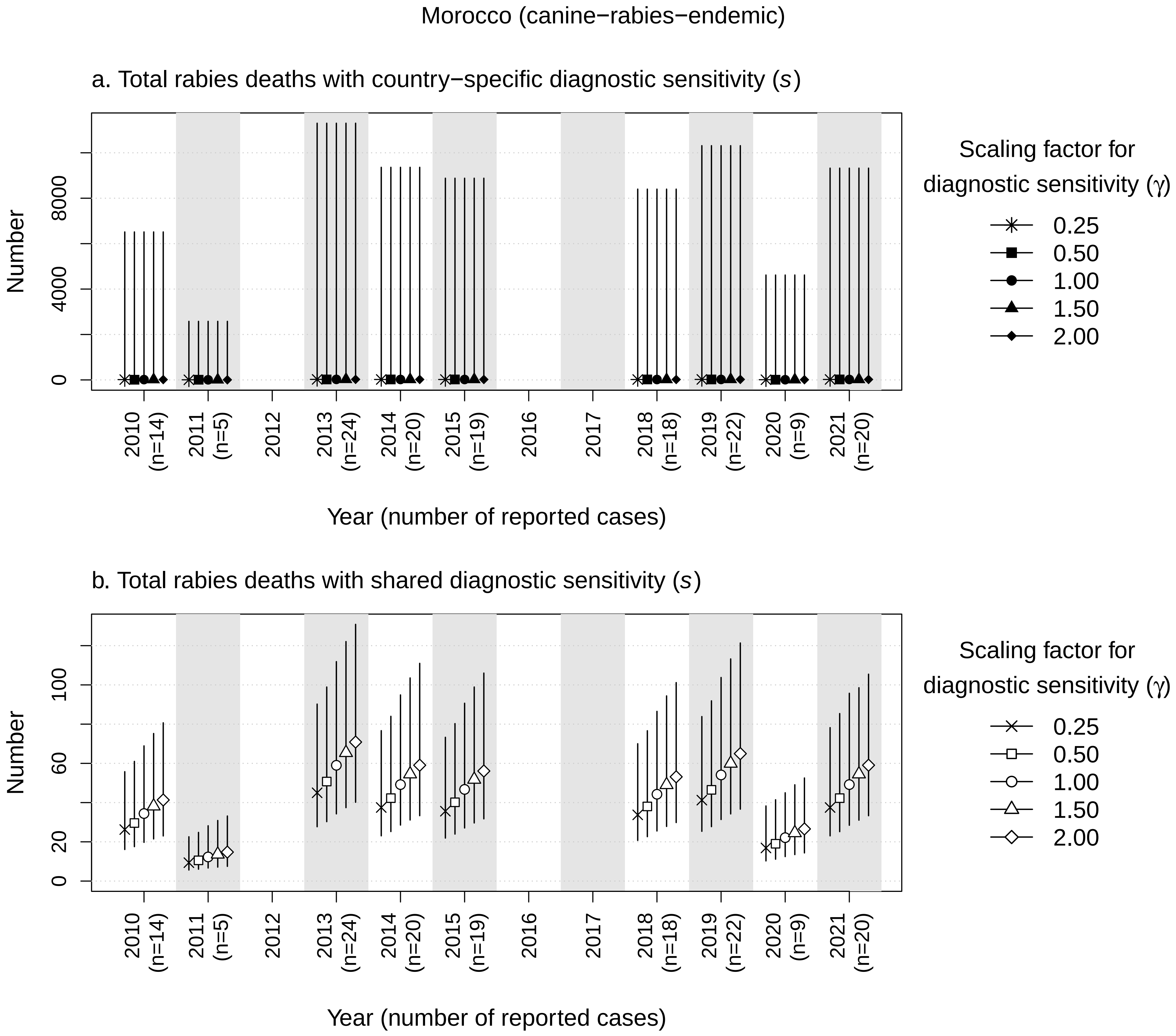
Morocco (canine−rabies−endemic)
a. Total rabies deaths with country−specific diagnostic sensitivity (s)
Number
0
4000
8000
Year (number of reported cases)
2010 (n=14)
2011 (n=5)
2012
2013 (n=24)
2014 (n=20)
2015 (n=19)
2016
2017
2018 (n=18)
2019 (n=22)
2020 (n=9)
2021 (n=20)
Scaling factor for diagnostic sensitivity (γ)
0.25
0.50
1.00
1.50
2.00
b. Total rabies deaths with shared diagnostic sensitivity (s)
Number
0
20
60
100
Year (number of reported cases)
2010 (n=14)
2011 (n=5)
2012
2013 (n=24)
2014 (n=20)
2015 (n=19)
2016
2017
2018 (n=18)
2019 (n=22)
2020 (n=9)
2021 (n=20)
Scaling factor for diagnostic sensitivity (γ)
0.25
0.50
1.00
1.50
2.00
